# CHILDHOOD ADVERSITY, NEIGHBORHOOD CONTEXTS, AND COGNITIVE FUNCTION IN ADULTHOOD

**DOI:** 10.1093/geroni/igad104.1765

**Published:** 2023-12-21

**Authors:** Jean Choi, Elizabeth Muñoz, Stacey Scott, Martin Sliwinski

**Affiliations:** The University of Texas at Austin, Austin, Texas, United States; The University of Texas at Austin, Austin, Texas, United States; Stony Brook University, Stony Brook, New York, United States; Pennsylvania State University, State College, Pennsylvania, United States

## Abstract

Childhood adversity may play an important role in cognitive health and aging. Neighborhood contexts in adulthood can serve as risks or resources that may modify the effects of childhood adversity on adult cognitive function, but these effects are not well-understood. We examined whether current positive (i.e., neighborhood cohesion) and negative (i.e., neighborhood violence) neighborhood contexts moderated the effect of childhood stress exposure on cognitive function in adulthood. We used data from 210 adults (Mage = 46.79, SD = 11.02; range: 25–65) residing in a single Zip code in Bronx, New York who completed measures of childhood adversity, current neighborhood contexts, and lab-based cognitive tasks. After adjusting for relevant demographic covariates, childhood adversity was not associated with cognitive function. The interaction between childhood adversity and neighborhood violence was significant, whereby lower childhood adversity was linked with worse performance in perceptual speed for individuals who reported both low (b = 1.19, SE = 0.37), p = .001) and high neighborhood violence (b = 0.75, SE = 0.26, p = .005), with higher scores indicating slower responses and worse performance. Unexpectedly, neighborhood violence was not predictive for those who reported greater childhood adversity. The interaction effect for neighborhood cohesion was not significant. Results indicate that childhood adversity may not have universal effects across all cognitive domains, but rather its effects are complex and may affect cognitive functioning to be adaptive to certain environmental contexts. This study underscores the need to replicate and examine the long-term consequences of these effects on cognitive aging.

